# Case report: Fibroadenomas associated with atypical ductal hyperplasia and infiltrating epitheliosis mimicking invasive carcinoma

**DOI:** 10.3389/fonc.2024.1354152

**Published:** 2024-03-08

**Authors:** Li Wang, Wei Zhao, Jue Zhou, Rong Ge

**Affiliations:** Department of Pathology, Ningbo Clinical Pathology Diagnosis Center, Ningbo, China

**Keywords:** breast, infiltrating epitheliosis, fibroadenoma, carcinoma, MEC

## Abstract

Infiltrating epitheliosis (IE) is an uncommon type of complex sclerosing lesion in the breast. This condition is characterized by the infiltration of ducts into a scleroelastotic stroma, along with the presence of cells that display architectural and cytological patterns similar to those observed in usual ductal hyperplasia. We herein report a case of a 24-year-old woman who presented with bilateral breast nodules, which were initially identified as multiple fibroadenomas based on ultrasound findings. The patient underwent Mammotome system and regional mastectomy procedures, and subsequent pathological analysis confirmed the presence of multiple fibroadenomas with atypical ductal hyperplasia and infiltrating epitheliosis. This case discusses the challenges faced in diagnosing malignancy in a patient with multiple fibroadenomas accompanied by atypical ductal hyperplasia and infiltrating epitheliosis.

## 1 Introduction

Fibroadenoma is a biphasic tumor composed of epithelial and stromal components, and it is the most common type of benign breast tumor that often presents in young women. However, in approximately half of the cases, fibroadenoma exhibits proliferative changes such as sclerosing adenosis, papillary apocrine metaplasia, and epithelial calcifications, which classify it as a complex fibroadenoma and a long-term risk factor for breast cancer (1).

Infiltrating epitheliosis (IE) is a rare complex sclerosing lesion of the breast, characterized by infiltrating ducts immersed in a scleroelastotic stroma and filled with cells exhibiting architectural and cytological patterns reminiscent of usual ductal hyperplasia (2). Due to limited experience with this phenomenon, we present an unusual case of fibroadenomas with atypical ductal hyperplasia and IE and analyze the challenges in diagnosis to minimize misdiagnosis and mistreatment.

## 2 Case presentation

A 24-year-old woman presented with complaints of bilateral mammary gland nodules that had been present for 1 month. She had no family history of breast cancer or ovarian lesions. Conventional laboratory investigations, including complete blood cell count, biochemical analysis, and serum levels of carcinoembryonic antigen, did not reveal any abnormalities.

**Figure 1:**
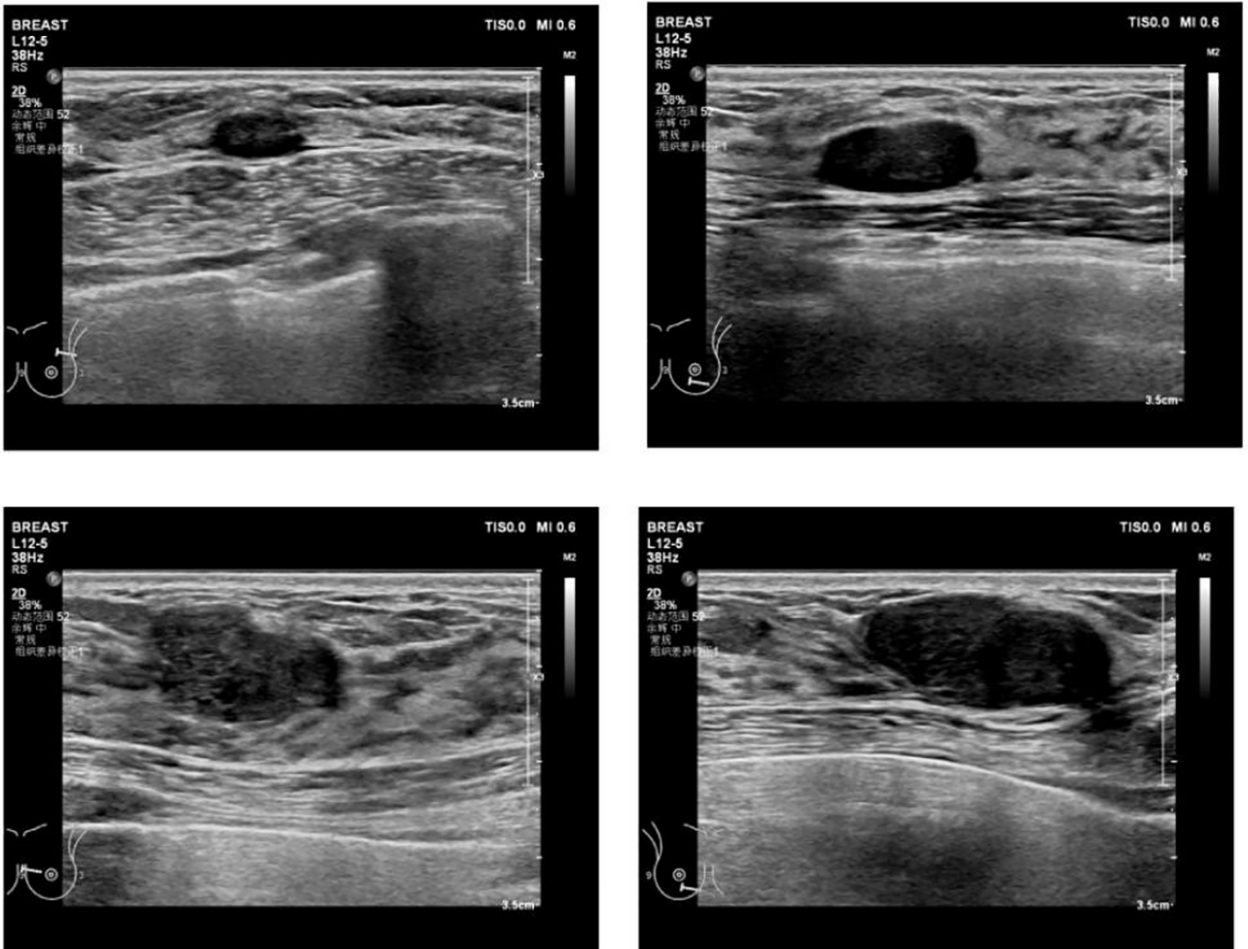
Ultrasound(US): bilateral mammary glands were irregular and flaky, local tissues were thickening, and there is structural disorder. There are several regular-shaped and well-circumscribed hypoechoic masses with envelope in her both breasts. CDFI: there was no obvious blood flow signal. Bilateral axillary lymph nodes were not palpable.

Ultrasound (US) examination revealed multiple hypoechoic masses with clear borders and smooth margins (as shown in **Figure 1**). The masses measured 1.1cm×0.5cm, 1.6cm×0.5cm, 1.3cm×0.6cm, 1.9cm×0.8cm, 1.8cm×0.8cm, 1.7cm×1.2cm, 2.2cm×1.3cm, and 2.2cm×0.8cm in the left breast at the positions of 2 o’clock, 6 o’clock, 9 o’clock, and 10 o’clock, and 1.4cm×0.7cm, 1.2cm×0.5cm, 2.7cm×1.3cm, 1.6cm×0.8cm, and 2.1cm×0.9cm in the right breast at the positions of 12 o’clock, 3 o’clock, 4 o’clock, 6 o’clock, and 11 o’clock. Color Doppler flow imaging (CDFI) did not show any obvious blood flow signals. Additionally, there were no palpable bilateral axillary lymph nodes.

**Figure 2:**
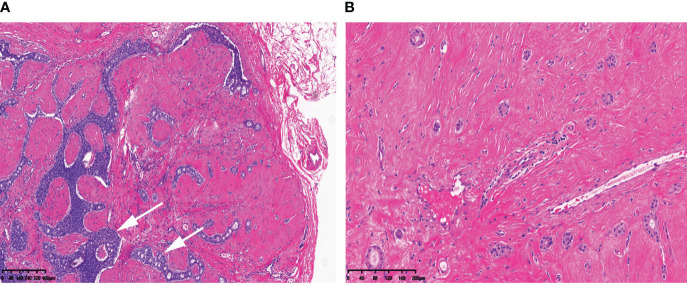
Morphological features: **(A)** one of the fibroadenomas in the right breast with intra-ductal epithelial hyperplasia, partly monoclonal sieve-like hyperplasia (arrows). **(B)** Several scattered tubules are observed in the surrounding stroma. Scale bars: **(A)** 80 μm; **(B)** 40 μm.

This patient is preparing for pregnancy and requests surgical removal of the breast mass in preparation for breastfeeding after childbirth. She underwent an excisional biopsy, and the frozen section analysis revealed characteristics consistent with fibroadenoma. The breast regional mastectomy was performed using the Mammotome system and regional mastectomy procedures. Routine sections of the tissue samples showed that almost all of the nodules were conventional fibroadenomas, except for one nodule in the right breast that exhibited atypical ductal hyperplasia (as shown in **Figure 2A**).

**Figure 3:**
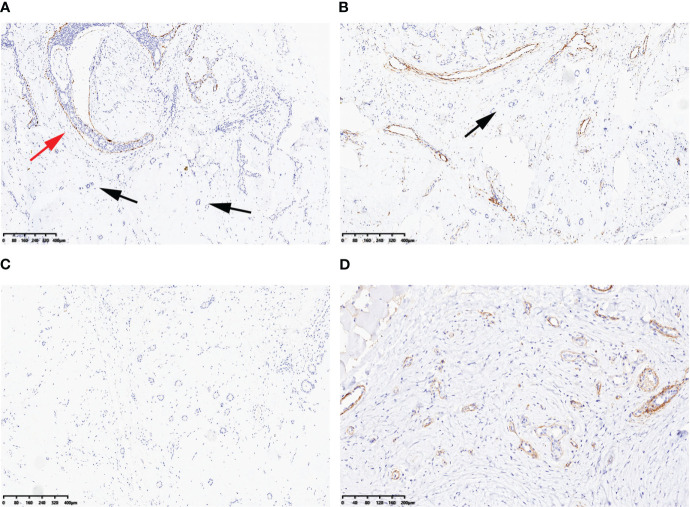
Immunohistochemical findings: CK14 **(A)** and Calponin **(B)** were preservative at the periphery of atypical ductal hyperplasia (red arrow), but were partly absent at scattered tubules in the surrounding stroma (black arrows). S100 protein was negative **(C)**. Collagen IV evidenced a continuous layer of basal lamina surrounding the glands **(D)**. Scale bars: **(A–C)** 80 μm; **(D)** 40 μm.

In the surrounding stroma, there were several scattered tubules that were partially absent in the myoepithelium (as shown in **Figure 2B**). Immunohistochemical staining for P63, SMA, CK5/6, and CK14 (as shown in **Figure 3A**) and Calponin (as shown in **Figure 3B**) confirmed this finding. The estrogen receptor (ER) was 2+ (80% positive), progesterone receptor (PR) was 3+ (90% positive), and human epidermal receptor-2 (HER-2) showed a membranous reaction of 0+. S100 protein staining was negative (as shown in **Figure 3C**). The Ki-67 index, which indicates the proliferative activity of cells, was <5%. Collagen IV evidenced a continuous layer of basal lamina surrounding the glands (as shown in **Figure 3D**). Therefore, the final diagnosis is multiple fibroadenomas with atypical ductal hyperplasia and IE.

## 3 Discussion

IE is a non-malignant breast lesion characterized by an infiltrative growth pattern and focal absence of myoepithelial cells (MECs) (3). It was initially described by John Azzopardi (4) in 1979 and referred to as “sclerosing adenosis with pseudo-infiltration” (5) or “sclerosing papillary proliferation” (6). Although its classification remains controversial, most pathologists currently classify IE within the spectrum of radial scar or complex sclerosing lesion (RS/CSL) (2).

Recent studies have suggested that IE may be neoplastic rather than hyperplastic and that the PI3K pathway is involved in its pathogenesis (2). The PI3K pathway plays crucial roles in cell survival, proliferation, and migration, and alterations in this pathway have been observed in 25%–50% of breast carcinomas (7), particularly low-grade and estrogen-receptor-positive carcinomas (8). Katie et al. reported that the frequency of PIK3CA mutations in RS was notably higher than the 25%–30% mutation frequency of invasive breast cancer (9). It has been indicated by Carey et al. that IE lesions may serve as a substrate from which ductal carcinoma *in situ* and low-grade adenosquamous carcinoma can originate. Furthermore, a subset of these lesions may have the potential to progress to invasive cancer (2).

In this case, the ultrasound images of fibroadenoma with IE cannot be distinguished from the other common fibroadenomas because all the nodules of preoperative ultrasound imaging presented with regular-shaped and well-circumscribed hypoechoic masses. Regrettably, the surgically removed nodules are not marked accordingly so that the differences of ultrasound imaging between it and other nodules could not be retrospectively analyzed based on pathological diagnosis.

Pathologically, IE can be easily mistaken for invasive carcinoma due to its infiltrative growth pattern with focal absence of myoepithelial cells (MECs). The histological characteristics of IE, as described by Eusebi and Millis (10), include the following: (a) the bulk of the lesion is composed of florid epitheliosis, often with a focal squamoid appearance; (b) scleroelastotic stromal changes are seen throughout the lesion, adjacent to epithelial foci, rather than confined to a central scleroelastotic nidus as observed in radial scars or complex sclerosing lesions; and (c) the frequent presence of a desmoplastic stromal reaction and keloid-like fibrous bands. Additionally, the involved ducts often exhibit irregular or jagged edges, and the proliferating epithelium often appears to “flow out” into the adjacent stroma (3, 11). Failure to recognize these exceptions such as IE and microglandular adenosis (MGA) can potentially result in incorrect classification as an invasive carcinoma, particularly tubular carcinoma (10–12) (**Table 1**).

**Table 1:** Differences and similarities of differential diagnosis.

	IE	MGA	TC
Glandular distribution	Irregular	Random	Wild
Shape of glands	Jagged edges	Round	Irregular to angulated
Stroma	Desmoplastic	Hyline/fibrofatty	Desmoplastic
Luminal secretion	Absent	Amorphous eosinophilic material	Absent
1 cell layer	Yes	Yes	Variable
MEC IHC	Absent	Absent	Absent
BL (Coll IV, Laminin)	Present	Present	Absent
S100	Absent	Present	Absent

IE, infiltrative epitheliosis; MGA, microglandular adenosis; TC, tubular carcinoma; MEC, myoepithelial cell; IHC, immunohistochemistry; BL, basal lamina; Coll IV, collagen IV.

Invasion is typically defined by the absence or breach of the basement membrane (BM) barrier between malignant epithelial cells and the surrounding stroma (3). However, these benign lesions that lack myoepithelial cells (MECs) exhibit infiltrating single-layer glands surrounded by a well-developed layer of BM (3). Due to the simplicity of immunohistochemical identification of the MEC layer, it is often used as a surrogate marker for invasion through the BM. It is important to note that MECs are predominantly lost at the periphery of IE, with frequent preservation at the epithelial–stroma interface in the center (3). In other words, MECs may be present in the proximal part of a duct but absent in the distal part.

On the other hand, MGA glands are lined by a single layer of cuboidal epithelial cells surrounded by a basal lamina, without any evidence of interposed MECs (13, 14). However, the lumens of this lesion often contain an amorphous eosinophilic material (11). Cells of MGA typically exhibit positive staining for low-weight keratins and S100 protein, but they are negative for estrogen receptor, progesterone receptor, and HER-2 (15). Therefore, the absence of MECs is not used as a criterion for diagnosing invasion because it can be observed in both benign and malignant lesions.

The management of infiltrative epitheliosis (IE) should follow the same approach as that of other complex sclerosing lesions so far (2). Additionally, careful monitoring is essential due to the risk of malignant transformation. Given the rarity of IE and the limited available data, further studies are necessary to understand its clinical behavior and to define the most appropriate surgical treatment.

In conclusion, infiltrative epitheliosis (IE) with an infiltrative growth pattern is a rare complex sclerosing lesion of the breast that can be mistaken for invasive carcinoma due to the absence of myoepithelial cells. Although IE is not routinely recognized in breast pathology practice at present, it does exist within the spectrum of breast lesions. Recognition of this rare lesion is important not only as an academic exercise but also for advancing our clinical understanding of IE, which may provide an ideal platform for studying the molecular mechanisms involved. Further studies are needed to better understand the clinical behavior of IE and to define appropriate management strategies for this rare breast lesion.

## Data availability statement

The original contributions presented in the study are included in the article/supplementary material. Further inquiries can be directed to the corresponding author.

## Ethics statement

The studies involving humans were approved by Ningbo Clinical Pathology Diagnosis Center. The studies were conducted in accordance with the local legislation and institutional requirements. The participants provided their written informed consent to participate in this study. Written informed consent was obtained from the individual(s) for the publication of any potentially identifiable images or data included in this article.

## Author contributions

LW: Writing – original draft. WZ: Data curation, Writing – review & editing. JZ: Writing – review & editing. RG: Writing – review & editing.
